# Synchrotron-based X-ray absorption near-edge spectroscopy imaging for laterally resolved speciation of selenium in fresh roots and leaves of wheat and rice

**DOI:** 10.1093/jxb/erv254

**Published:** 2015-05-26

**Authors:** Peng Wang, Neal W. Menzies, Enzo Lombi, Brigid A. McKenna, Simon James, Caixian Tang, Peter M. Kopittke

**Affiliations:** ^1^The University of Queensland, School of Agriculture and Food Sciences, St. Lucia, Queensland, 4072, Australia; ^2^University of South Australia, Centre for Environmental Risk Assessment and Remediation, Mawson Lakes, South Australia, 5095, Australia; ^3^Australian Synchrotron, Clayton, Victoria, 3168, Australia; ^4^La Trobe University, Centre for AgriBioscience, Bundoora, Victoria, 3086, Australia

**Keywords:** Fluorescence-XANES imaging, laterally resolved speciation, selenium uptake, speciation, transformation, translocation.

## Abstract

Knowledge of the distribution of selenium (Se) species within plant tissues will assist in understanding the mechanisms of Se uptake and translocation, but *in situ* analysis of fresh and highly hydrated plant tissues is challenging. Using synchrotron-based fluorescence X-ray absorption near-edge spectroscopy (XANES) imaging to provide laterally resolved data, the speciation of Se in fresh roots and leaves of wheat (*Triticum aestivum* L.) and rice (*Oryza sativa* L.) supplied with 1 μM of either selenate or selenite was investigated. For plant roots exposed to selenate, the majority of the Se was efficiently converted to C-Se-C compounds (i.e. methylselenocysteine or selenomethionine) as selenate was transported radially through the root cylinder. Indeed, even in the rhizodermis which is exposed directly to the bulk solution, only 12–31% of the Se was present as uncomplexed selenate. The C-Se-C compounds were probably sequestered within the roots, whilst much of the remaining uncomplexed Se was translocated to the leaves—selenate accounting for 52–56% of the total Se in the leaves. In a similar manner, for plants exposed to selenite, the Se was efficiently converted to C-Se-C compounds within the roots, with only a small proportion of uncomplexed selenite observed within the outer root tissues. This resulted in a substantial decrease in translocation of Se from the roots to leaves of selenite-exposed plants. This study provides important information for understanding the mechanisms responsible for the uptake and subsequent transformation of Se in plants.

## Highlight

Using synchrotron-based XANES imaging, *in situ* laterally resolved speciation of Se in hydrated tissues was obtained, which assists in understanding the Se uptake, translocation, and transformation in fresh plants.

## Introduction

Selenium (Se) is an essential micronutrient (Rayman, 2000) in which up to 1 billion people worldwide are deficient (Combs, 2001). A recent global survey of grains of rice (*Oryza sativa* L.) showed that 75% of the grain had an Se concentration insufficient for human requirements (Williams *et al.*, 2009). Plant breeding strategies (i.e. genetic biofortification) and application of Se fertilizers (i.e. agronomic biofortification) have been proposed as an effective way of increasing Se intake in humans (Hawkesford and Zhao, 2007). Understanding the mechanisms of Se uptake and accumulation in plants would help improve the efficiency of biofortification programmes and direct the breeding of cultivars with higher Se concentrations in grains.

Selenite [Se(IV)] and selenate [Se(VI)] are the two dominant inorganic species of Se in soils. The mechanism of Se(VI) uptake by plant roots is well known—it is taken up via the high affinity sulphate transporters (Terry *et al.*, 2000; Sors *et al.*, 2005). After its uptake, the major form for translocation is Se(VI) (i.e. without chemical modification). However, this translocation of Se(VI) is limited by its comparatively efficient reduction within the root to Se(IV) via ATP sulphurylase. Through a selenocysteine methyltransferase (SMT), the intermediate Se(IV) is further assimilated into selenocysteine (SeCys), which is the precursor of organic Se compounds (Terry *et al.*, 2000). The former is often the rate-limiting step in the metabolism of Se(VI) (de Souza *et al.*, 1998).

In contrast, little is known about the mechanisms involved in Se(IV) uptake. It has been suggested that Se(IV) is taken up through passive diffusion (Shrift and Ulrich, 1969; Arvy, 1993), phosphate transporters (Broyer *et al.*, 1972; Hopper and Parker, 1999; Li *et al.*, 2008; Zhang *et al.*, 2014), and/or a silicon (Si) influx transporter Lsi1 (OsNIP2;1) (Zhao *et al.*, 2010). Apart from the differences in their mechanisms of uptake, Se(IV) and Se(VI) also differ in their mobility within plants (Sors *et al.*, 2005; Li *et al.*, 2008; Yu and Gu, 2008; Wang *et al.*, 2013*b*). Indeed, Se(IV) is efficiently converted to organic forms in roots, which limits translocation to shoots. In contrast, Se(VI) is highly mobile in xylem transport, with only a relatively small proportion assimilated to organic forms in roots (Bulska *et al.*, 2006; Li *et al.*, 2008; Wang *et al.*, 2013*b*).

These changes in Se speciation discussed above have often been measured in bulk tissues, and to the authors’ knowledge only a single study that has provided *in situ* laterally resolved data regarding the speciation of Se in a hyperaccumulator, *Astragalus bisulcatus* (Pickering *et al.*, 2000). However, in the study of Pickering *et al.* (2000), maps were collected at only the two energies (12.661 keV and 12.667 keV) corresponding to the peaks of the two Se compounds of interest, with the acquisition time being 10 s per pixel. Accurate determination of speciation based upon only two energies is difficult, especially when there are overlaps in their spectra. Also, long acquisition times increase the likelihood of changes in speciation resulting from photoreduction damage induced by the X-ray beam, as has been observed previously in studies investigating Se (Wang *et al.*, 2013*b*). Finally, it is noteworthy that this study of Pickering *et al.* (2000) was conducted using a hyperaccumulator— studies with agronomic species are more difficult due to their substantially lower tissue concentrations.

The Maia detector system is one of several new-generation X-ray fluorescence (XRF) detectors, and is operational at the Australian Synchrotron (Ryan *et al.*, 2010). Due to its capability of faster data acquisition, fluorescence X-ray absorption near-edge spectroscopy (XANES) imaging has been developed as an analytical technique to obtain laterally resolved speciation mapping by scanning the same area repeatedly with increasing (or decreasing) energies across an absorption edge. This approach differs from earlier studies where a map is obtained and then XANES spectra are collected from specific regions of interest within that map. With XANES imaging, XANES spectra are collected for each pixel of the entire map, thus permitting more informative chemical analyses (e.g. Kopittke *et al.*, 2014).

Using this Maia detector system installed at the XFM beamline at the Australian Synchrotron, it was possible to collect fluorescence maps at 86 energies across the Se absorption edge, allowing a much more informative analysis from the spectra obtained. This increase in the number of energies examined in the present study was possible due to the decreased dwell required with the Maia detector, with <8ms required per pixel per energy (i.e. <0.69 s per pixel for 86 energies). The increased sensitivity of the Maia detector (i.e. decreased dwell) also minimizes the risk of photoreduction damage.

This study aimed to utilize fluorescence-XANES imaging to investigate the *in situ* laterally resolved speciation of Se within hydrated and fresh roots and leaves of wheat (*Triticum aestivum* L.) and rice supplied with either Se(VI) or Se(IV). These data provide information regarding the accumulation and translocation of Se within fresh plant tissues, thus assisting in understanding the mechanism for Se uptake and transformation in plants.

## Materials and methods

### Plant growth

Seeds of wheat (cv. Sunbrook) and rice (cv. Nipponbare) were germinated in trays covered with paper towels moistened with tap water. The temperature was maintained at 25 °C in a laboratory at The University of Queensland (St Lucia, Australia) for 3 d for wheat or 4 d for rice. The seedlings were transferred to containers with 11 litres of nutrient solution (see details below). Each container was covered by a plastic lid with four holes. Five seedlings were transplanted to each of the four holes, with the seedlings suspended using shade cloth and polypropylene beads used to limit entry of light into the nutrient solution. The plants were grown at 25 °C under alternate 16h of light (photon flux density of 500 μmol m^–2^ s^–1^) and 8h of darkness.

A nutrient solution was prepared with the following final composition (μM): 800 N (supplied as 680 NO_3_^–^ and 120 NH_4_^+^), 100 Ca, 100 Mg, 310 K, 50 S, 564 Cl, 10 P, 200 Fe [supplied as Fe(III)EDTA], 3.0 B, 1.0 Mn, 0.05 Cu, 1.0 Zn, and 0.02 Mo. The pH value of the continuously aerated nutrient solution was not adjusted (initial pH 5.6) and solutions were renewed every 2 d. After a further 7 d, Se stock solutions (6.5mM Na_2_SeO_3_ or Na_2_SeO_4_) were added to yield a final concentration of 1 μM of either Se(IV) or Se(VI). The plants were grown in this solution for 6 d before transfer to the Australian Synchrotron (Melbourne, Australia) for analysis. Once at the Australian Synchrotron, the plants were grown under the same conditions and placed in individual containers (2 litres) filled with continuously aerated Se-containing nutrient solution for a further 1–2 d before analysis by XRF microscopy (μ-XRF). In addition, subsamples were collected to measure bulk concentrations of Se in plant tissues. Briefly, ~1–2g of plant tissues were digested with 5ml of acid (5:1 nitric acid:perchloric acid). Following digestion, the samples were diluted to 10ml using deionized water before analysis using inductively coupled plasma mass spectrometry (ICP-MS).

### μ-XRF and fluorescence-XANES imaging

Following exposure to Se, roots and leaves were harvested with the apical 5–10mm of each root/leaf segment placed between two pieces of 8 μm thick Kapton polyimide film, which formed a tight seal around the roots/leaves to minimize dehydration. For each plant species, at least three replicate roots and two replicate leaves from individual plants were examined for each treatment, with the replicated roots and leaves positioned vertically in the sample holder and scanned simultaneously. Samples were prepared and examined at the X-ray fluorescence microscopy (XFM) beamline at the Australian Synchrotron, where an in-vacuum undulator is used to produce a brilliant X-ray beam. An Si(111) monochromator and Kirkpatrick–Baez mirrors are used to obtain a monochromatic beam focused onto the specimen (Paterson *et al.*, 2007, 2011). The X-ray fluorescence emitted by the specimen was collected using the 384 element Maia detector placed in a backscatter geometry (Kirkham *et al.*, 2010). For all scans, samples were analysed continuously in the horizontal direction (‘on the fly’).

For plant roots or leaves, an initial large area survey scan (‘pre-XANES survey scan’) was conducted in order to identify the area of interest and to obtain overall elemental distributions. Subsequently, a smaller area was chosen to conduct the fluorescence-XANES imaging (‘XANES imaging scan’), with the XANES stack itself consisting of 86 individual maps at decreasing energies (see below). Finally, a repeat survey scan (‘post-XANES survey scan’) was conducted to allow a comparison with the first scan to identify potential beam damage as evident by changes in elemental distribution.

For the survey scans, the beam was focused to either 2.6 μm wide (horizontal direction) by 5 μm high (vertical direction) for roots or 2.6 μm×10 μm for leaves, with an energy of 15.6 keV. The specimen was scanned through the focus in the horizontal direction and the fluorescence event stream divided into ‘virtual pixels’ either 8 μm (roots) or 15 μm (leaves) in width. At the end of each line, the specimen was stepped by 8 μm (roots) or 50 μm (leaves) in the vertical direction. The scanning velocity was either 0.512mm s^–1^ or 1.024mm s^–1^ for root samples and 8.192mm s^–1^ for leaf samples. Thus, the total time for each survey map was ~20–40min depending on the size of the area of interest.

For the fluorescence-XANES imaging, the focused beam size was 2.6 μm×5 μm for root samples with a 10 μm sampling interval in the vertical direction and a 2 μm sampling interval in the horizontal direction. The transit time per pixel was 7.81ms (0.256mm s^–1^ scanning velocity) and hence the total scan time (three replicate roots for each treatment) for the 86 energies in the XANES stack was ~4h. For leaf samples, the beam was focused to 2.6 μm×10 μm with a 15 μm sampling interval in the horizontal direction and a 50 μm sampling interval in the vertical direction. The transit time per pixel was 0.97ms (2.048mm s^–1^ scanning velocity) and the total scan time (two leaves per species for each treatment) for the 86 energies in the XANES stack was ~3.5h.

The fluorescence-XANES imaging (i.e. XANES imaging scan) consisted of ‘stacks’ of 86 μ-XRF maps collected at decreasing incident energies from 12.905 keV to 12.587 keV across the Se K-edge. In order to match the energy location of the features of the Se XANES spectra of the different Se species (i.e. inorganic or organic Se), the energies of these 86 progressive scans were selected as follows: 12.905–12.802 in 0.02 keV decrements (six energies), 12.792–12.702 in 0.01 keV decrements (10 energies), 12.699–12.672 in 0.003 keV decrements (10 energies), 12.671–12.652 in 0.0005 keV decrements (40 energies), and 12.651–12.637 in 0.001 keV decrements (15 energies).

Five standard compounds were also analysed as solutions using fluorescence-XANES imaging: Na_2_SeO_4_ (Sigma-Aldrich; S0882), Na_2_SeO_3_ (Sigma-Aldrich; 2114485), SeCys (Sigma-Aldrich; 545996), selenomethionine (SeMet, Sigma-Aldrich; S3875), and methylselenocysteine (MeSeCys, Sigma-Aldrich; M6680). All standards were prepared to a final Se concentration of 1.3mM. For each standard solution, a strip of filter paper (~3mm wide) was immersed for 3 s. The five strips were wrapped in polyimide film and placed vertically on a sample holder for analysis using fluorescence-XANES imaging.

In all instances, the μ-XRF spectra were analysed using GeoPIXE (Ryan *et al.*, 2005) and the images were generated using the Dynamic Analysis method (Ryan *et al.*, 2010). For the fluorescence-XANES stacks, the GeoPIXE ‘energy association’ module was used to compare the concentration ratios between two energies. Specifically, pixel populations were selected where either the relative proportions of the various Se species varied and/or the absolute concentrations of the Se species varied. For example, in Supplementary Fig. S1B available at *JXB* online, the pixels are generally not parallel to the 1:1 line (particularly at the highest Se concentrations), and hence the pixel populations selected represented not only a change in their absolute concentrations but also a change in the proportion (ratio) of Se species. However, for the data presented in Supplementary Fig. S2B, the pixels were generally parallel to the 1:1 line, and hence the pixel populations selected generally represent a change only in their absolute concentrations (whilst their proportions remain constant). From the selected pixel populations, potential changes in speciation were identified by extracting the XANES spectra which were then energy-normalized using Athena (Ravel and Newville, 2005). For these XANES spectra (–30 to 100 E, eV), linear combination fitting (LCF) was performed using Athena, with the combination of standards yielding the lowest residual parameter chosen as the most likely set of components. Areal concentrations of the various Se species were calculated by multiplying the areal Se concentration determined from the survey maps using GeoPIXE by their relative proportion determined by LCF. Projected volumetric concentrations of the Se species were determined by multiplying the areal concentration by the ratio of the thickness of the root at each point of the scan to the assumed effective thickness by GeoPIXE. Using these data, the concentration of each Se species within the individual tissues of the concentric root cylinder were calculated using a model described previously (Wang *et al.*, 2013*a*). Given that the pixel size was 10 μm, it was not possible to differentiate among the rhizodermis, exodermis, and sclerenchyma in rice, and hence these were examined as a single tissue. To estimate the Se concentration in the rhizodermis (C_1_, R_1_), cortex (C_2_, R_2_), and stele (C_3_, R_3_), the root radius R was first inputted by measuring the diameter of the root cylinder using GeoPIXE (for a detailed description, see Wang *et al.*, 2013*a*). R_1_ to R_3_ were initially assigned according to the ratios of transverse sections. To achieve the estimates, values for C_output_ were computed with equations (see Wang *et al.*, 2013*a*) using parameters to optimize the correspondence between the calculated C_output_ and the observed C_output_ produced by GeoPIXE. Optimization entailed the incorporation of starting values into an iterative program (SOLVER program in Microsoft Excel 2010) to compute C_output_.

### Statistical analysis

Treatment differences were tested for significance (*P*<0.05) using a one-way analysis of variance performed with IBM SPSS Statistics 20.

## Results

### Se accumulation within bulk plant tissues

The form of Se supplied within the nutrient solution had a substantial impact upon the accumulation and translocation of Se (Table 1). In particular, bulk tissue concentrations were higher in leaves than in roots for plants exposed to Se(VI), but the opposite was true for Se(IV)-exposed plants. For example, for rice plants grown at 1 μM Se(VI) for 1 week, the concentrations of Se were 6.25 μg g^–1^ (fresh mass basis) in the roots and 66.5 μg g^–1^ in the leaves, with an Se translocation factor (TF; i.e. the ratio of Se in the leaf to that in the root) of 10.6. In contrast, for rice plants exposed to 1 μM Se(IV), Se concentrations were 14.5 μg g^–1^ in the roots [2.3-fold higher than for Se(VI)] and 10.9 μg g^–1^ in the leaves [6.1-fold lower than for Se(VI)], with a TF of 0.75. Similar trends were observed for wheat seedlings (Table 1). Accordingly, ~89% of Se was translocated to the leaves in plants exposed to Se(VI) whereas only 43% was translocated to the leaves when exposed to Se(IV).

**Table 1.** Concentrations of Se in bulk tissues and the translocation factor (TF; i.e. the ratio of Se in the leaf to that in the root) of wheat and rice exposed to nutrient solution containing 1 μM of either Se(IV) or Se(VI) for 1 week All concentration are expressed on a fresh weight basis and are the arithmetic mean ±SE of four replicates.

Plants	Treatment	Se concentration (mg kg^–1^ FW)		TF
		Roots	Leaves	
Rice	Selenate	6.25±0.49 a	66.5±1.84 c	10.6
	Selenite	14.5±1.23 c	10.9±0.62 b	0.75
Wheat	Selenate	7.22±1.72 a	46.4±12.3 c	6.43
	Selenite	10.6±1.82 b	8.08±0.91 a	0.76

Within each column, means with different letters are significantly different (*P*<0.05, *t*-test).

### Se reference XANES spectra

Substantial differences were observed between the five standard compounds. For the two inorganic compounds (Supplementary Fig. S3 at *JXB* online), the white line shifted from 12.667 keV for Se(VI) to 12.664 keV for Se(IV). The organic Se compounds were characterized by a decreased intensity of the main fluorescence peak, with the white line for SeCys (selenocysteine) being 12.559 keV. As expected, the XANES spectra for MeSeCys and SeMet were quite similar, with white lines at 12.661 keV—this reduces the ability to distinguish between these two compounds in the present study. Therefore, where LCF analyses indicated that Se was present in plant tissues as one of these two forms (MeSeCys or SeMet), their contributions were summed and defined as ‘C-Se-C compounds’ (Tables 2–4).

**Table 2.** *Results of linear combination fitting (LCF) of Se K-edge XANES data for roots of rice (*Oryza sativa *L.) and wheat (*Triticum aestivum *L.) exposed to 1 μM of either Se(IV) or Se(VI) for 1 week* These analyses are based upon two-dimensional analyses of three-dimensional roots (for an example, see Fig. 1C, D) and do not take into account their concentric structure (Table 3).

	Rice						Wheat				
	Exposed to Se(IV)			Exposed to Se(VI)			Exposed to Se(IV)		Exposed to Se(VI)		
	Outer	Inner	Lateral root primordia	Outer	Middle	Inner	Outer	Inner	Outer	Middle	Inner
C-Se-C compounds^*a*^ (%)	94 (1.4)	99 (0.7)	99 (3.3)	69 (0.8)	77 (0.5)	84 (0.4)	96 (1.4)	100 (0)	88 (0.9)	90 (0.7)	92 (0.4)
Se(IV) (%)	6.3 (1.4)	1.1 (0.7)	1.5 (3.3)				3.6 (1.4)				
Se(VI) (%)				31 (0.8)	23 (0.5)	16 (0.4)			12 (0.9)	10 (0.7)	7.9 (0.4)
R-factor	0.0025	0.0012	0.0104	0.0033	0.0016	0.0006	0.0027	0.0021	0.0050	0.0027	0.0010

**Fig. 1.**
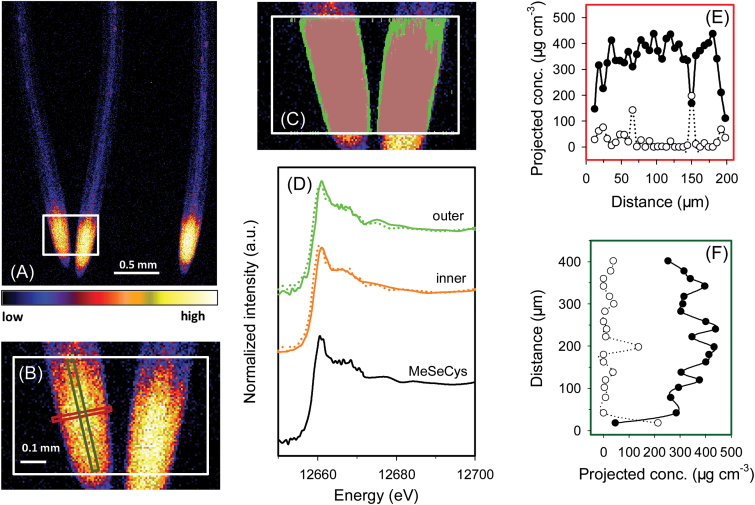
Rice (*Oryza sativa* L.) roots exposed to nutrient solution containing 1 μM Se(IV) for 1 week. (A and B) Elemental survey maps showing total Se distribution collected in the ‘pre-XANES survey scan’ followed by fluorescence-XANES imaging (‘XANES imaging scan’), with the white box (0.56 mm×0.40mm) indicating the area examined by XANES imaging. (C) The spatial distribution of two pixel populations (outer and inner) identified by comparing energy intensities. (D) Normalized Se K-edge XANES spectra corresponding to the two pixel populations ‘outer’ and ‘inner’ shown in (C) plus the spectrum for MeSeCys. (E and F) Projected volumetric concentrations of C-Se-C compounds (i.e. MeSeCys or SeMet, filled circles) and uncomplexed Se(IV) (open circles) in the latitudinal or longitudinal transects indicated by the red or green rectangle in (B). Dotted lines in (D) show the best fits of reference spectra obtained using LCF as presented in Table 2. Distance in (E) and (F) refers to distance from left to right in the red rectangle and from bottom to top in the green rectangle indicated in (B), respectively. The projected concentrations in (E) and (F) were obtained by multiplying the projected areal concentration by the calculated thickness of the root to obtain the projected volumetric concentration, and multiplying the projected volumetric concentration by the proportion of the Se species (determined for each point using linear combination fitting). Taking into account the concentric structure of roots, concentrations of the Se species in the individual root tissues were calculated in Table 3.

**Fig. 2.**
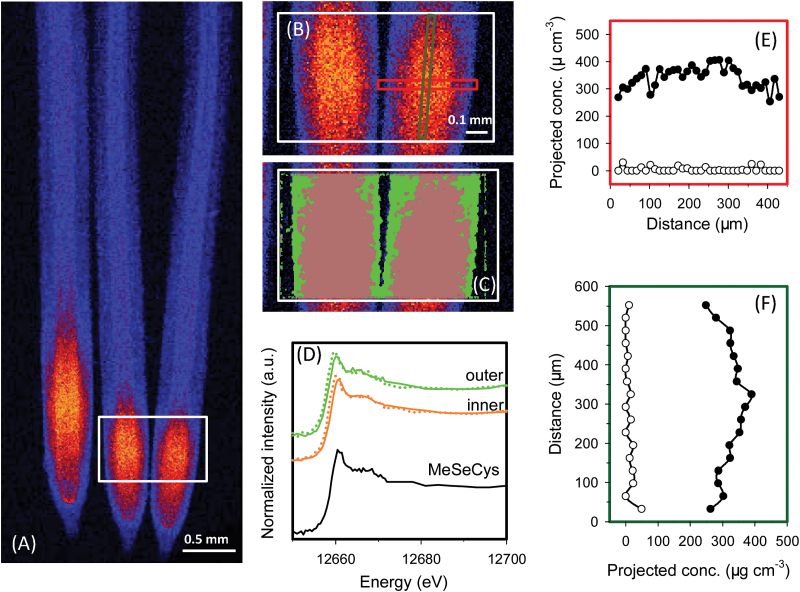
Wheat (*Triticum aestivum* L.) roots exposed to nutrient solution containing 1 μM Se(IV) for 1 week. A full description is given in the legend of Fig. 1. (A and B) Elemental survey maps showing total Se distribution collected in the ‘pre-XANES survey scan’ followed by fluorescence-XANES imaging (‘XANES imaging scan’), with the white box (0.97 mm×0.60mm) indicating the area examined by XANES imaging. (C) The spatial distribution of two pixel populations (outer and inner) identified by comparing energy intensities. (D) Normalized Se K-edge XANES spectra corresponding to the two pixel populations ‘outer’ and ‘inner’ shown in (C) plus the spectrum for MeSeCys. (E and F) Projected volumetric concentrations of C-Se-C compounds (i.e. MeSeCys or SeMet, filled circles) and uncomplexed Se(IV) (open circles) in the cross- and longitudinal transects indicated by the red or green rectangle in (B).

**Fig. 3.**
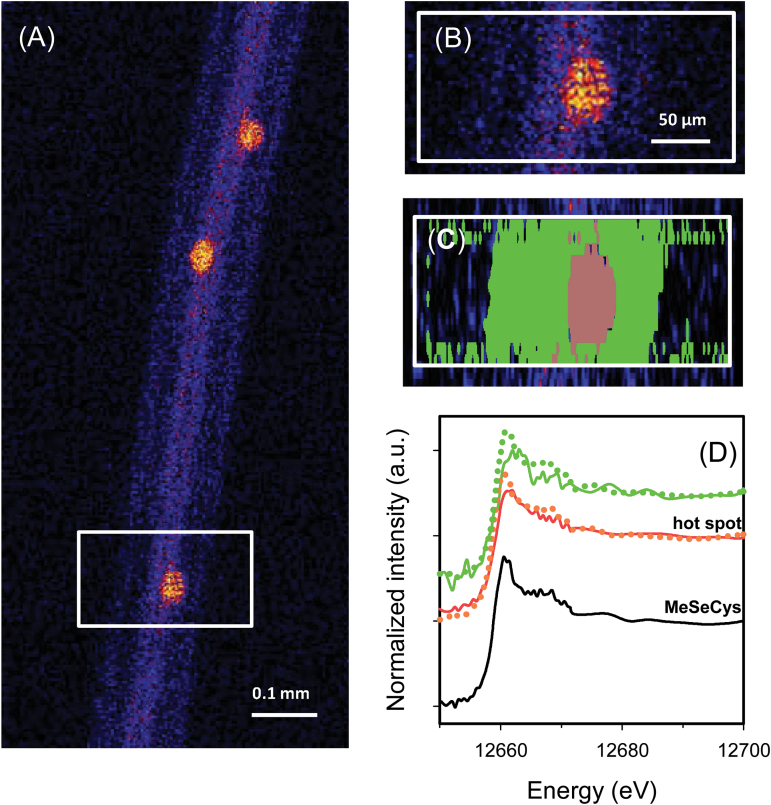
The mature primary root (3.0mm from the tip) of rice (*Oryza sativa* L.), with the lateral root primordium which initiates from the primary root. (A and B) Elemental survey maps showing total Se distribution collected in the ‘pre-XANES survey scan’ followed by fluorescence-XANES imaging (‘XANES imaging scan’), with the white box (0.27 mm×0.15mm) indicating the area examined by XANES imaging. (C) The spatial distribution of two pixel populations (outer tissues and hot spot) identified by comparing energy intensities. (D Normalized Se K-edge XANES spectra corresponding to the two pixel populations ‘hot spot’ and ‘outer tissues’ shown in (C) plus the spectrum for MeSeCys.

**Fig. 4.**
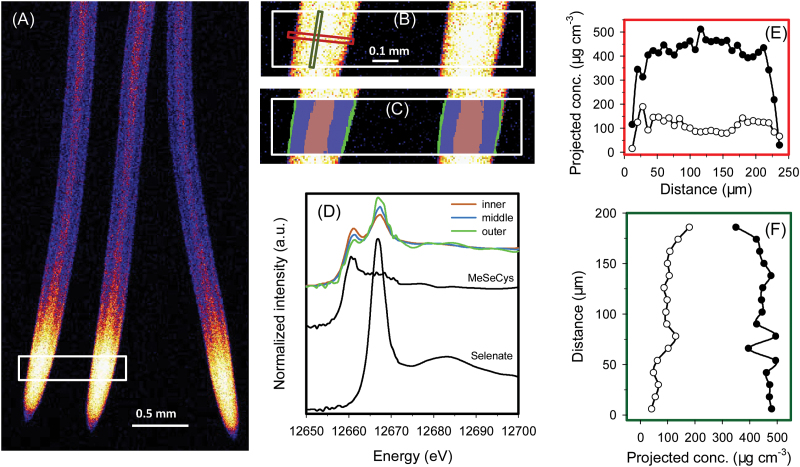
Rice (*Oryza sativa* L.) roots exposed to nutrient solution containing 1 μM Se(VI) for 1 week. A full description is given in the legend of Fig. 1. (A and B) Elemental survey maps showing total Se distribution collected in the ‘pre-XANES survey scan’ followed by fluorescence-XANES imaging (‘XANES imaging scan’), with the white box (0.95 mm×0.20mm) indicating the area examined by XANES imaging. (C) The spatial distribution of three pixel populations (outer, middle, and inner) identified by comparing energy intensities. (D) Normalized Se K-edge XANES spectra corresponding to the three pixel populations ‘outer’, ‘middle’, and ‘inner’ shown in (C) plus the spectrum for MeSeCys. (E and F) Projected volumetric concentrations of C-Se-C compounds (i.e. MeSeCys or SeMet, filled circles) and uncomplexed Se(VI) (open circles) in the cross- or longitudinal transects indicated by the red or green rectangle in (B).

**Fig. 5.**
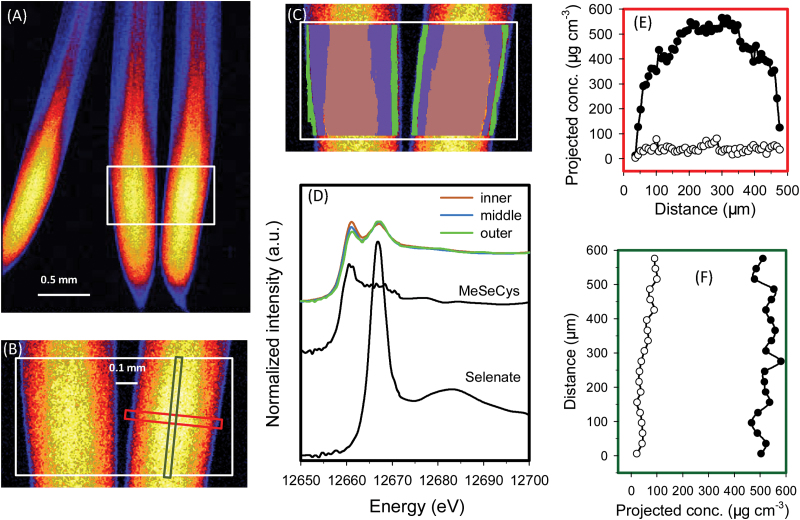
Wheat (*Triticum aestivum* L.) roots exposed to nutrient solution containing 1 μM Se(VI) for 1 week. A full description is given in the legend of Fig. 1. (A and B) Elemental survey maps showing total Se distribution collected in the ‘pre-XANES survey scan’ followed by fluorescence-XANES imaging (‘XANES imaging scan’), with the white box (0.97 mm×0.60mm) indicating the area examined by XANES imaging. (C) The spatial distribution of three pixel populations (outer, middle, and inner) identified by comparing energy intensities. (D) Normalized Se K-edge XANES spectra corresponding to the three pixel populations ‘outer’, ‘middle’, and ‘inner’ shown in (C) plus the spectrum for MeSeCys. (E and F) Projected volumetric concentrations of C-Se-C compounds (i.e. MeSeCys or SeMet, filled circles) and uncomplexed Se(VI) (open circles) in the cross- or longitudinal transects indicated by the red or green rectangle in (B).

Data are presented for the ‘outer’, ‘middle’, and ‘inner’ tissues (see Figs 1, 2, 4, 5) and the lateral root primordia (Fig. 3).

Data are rounded to two significant figures, means (SE). The spatial distribution of three pixel populations (outer, middle, and inner) identified by comparing energy intensities (see Fig. 1 and Supplementary Fig. S1 at *JXB* online for an example).

The R-factor is the residual factor generated by the LCF tool in Athena and indicates the goodness of fit, with R-factor=∑i(data–fit)^2^/∑i(data)^2^.

^*a*^ C-Se-C compounds refer to selenomethionine (SeMet) or methylselenocysteine (MeSeCys).

**Table 3.** *Projected volumetric concentrations of C-Se-C compounds (i.e. MeSeCys or SeMet), uncomplexed Se(IV), and uncomplexed Se(VI) within the rhizodermis (rhizo.), cortex, and stele of root tissues of wheat (*Triticum aestivum *L.) and rice (*Oryza sativa *L.) exposed to nutrient solutions containing 1 μM of either Se(IV) or Se(VI*)

	Rice						Wheat					
	Exposed to Se(IV)			Exposed to Se(VI)			Exposed to Se(IV)			Exposed to Se(VI)		
	Rhizo.	Cortex	Stele	Rhizo.	Cortex	Stele	Rhizo.	Cortex	Stele	Rhizo.	Cortex	Stele
C-Se-C compounds (μg cm^–3^)	355	301	539	413	507	632	324	397	434	441	671	584
Uncomplexed Se(IV) (μg cm^–3^)	21.2	62.1	0				8.0	0	3.4			
Uncomplexed Se(VI) (μg cm^–3^)				116	157	23.7				57	29	64

Concentrations were calculated using the mathematical model of Wang *et al.* (2013*a* ) from the projected volumetric concentrations across transects ~0.5mm from the apex (see Fig. 1E). The endodermis was not included in the calculations as it was not formed close to the tip. Given that the pixel size was 10 μm, it was not possible to differentiate among the rhizodermis, exodermis, and sclerenchyma in rice, and hence these were examined as a single tissue.

**Table 4.** *Result of linear combination fitting (LCF) of Se K-edge XANES data for leaves of both rice (*Oryza sativa *L.) and wheat (*Triticum aestivum *L.) grown for 1 week in nutrient solutions containing 1 μM of either Se(IV) or Se(VI*) The values are the arithmetic means of the two species.

	Exposed to Se(IV)		Exposed to Se(VI)	
	Inter-vein	Vein	Inter-vein	Vein
C-Se-C compounds^*a*^ (%)	100 (0)	100 (0)	44 (0.3)	48 (0.4)
Uncomplexed Se(IV) (%)				
Uncomplexed Se(VI) (%)			56 (0.3)	52 (0.4)
R-factor	0.0011	0.0035	0.0004	0.0008

**Fig. 6.**
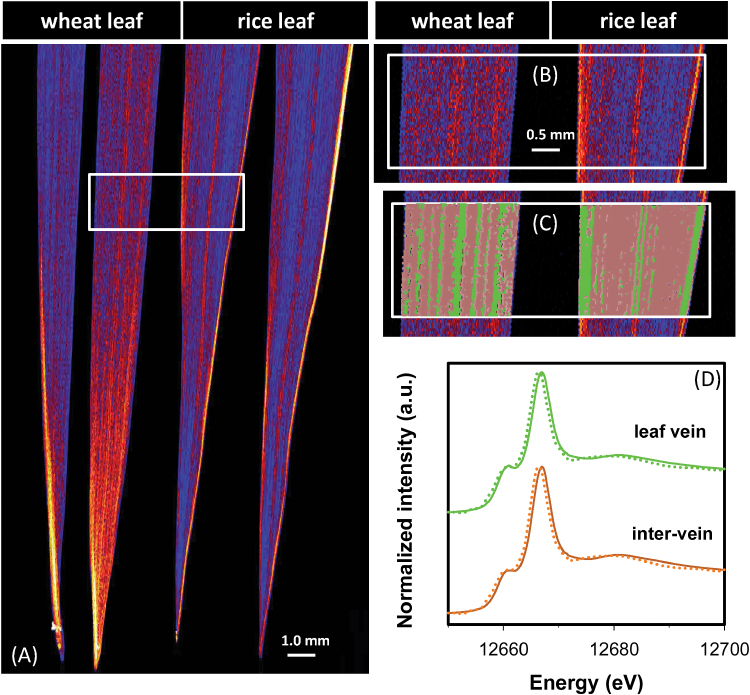
Leaves of wheat (*Triticum aestivum* L.) and rice (*Oryza sativa* L.) grown in nutrient solution containing 1 μM Se(VI) for 1 week. (A and B) Elemental survey maps showing total Se distribution collected in the ‘pre-XANES survey scan’ followed by fluorescence-XANES imaging (‘XANES imaging scan’), with the white box (5.6 mm×2.0mm) indicating the area examined by XANES imaging. (C) The spatial distribution of two pixel populations (leaf vein and inter-vein) identified by comparing energy intensities. (D) Normalized Se K-edge XANES spectra corresponding to the two pixel populations ‘leaf vein’ and ‘inter-vein’.

Data are presented for the ‘inter-vein’ and ‘vein’ tissues (see Fig. 6; Supplementary Fig. S7 at *JXB* online). The values in parentheses show the SE in the calculated values in LCF analysis.

The spatial distribution of two pixel populations (inter-vein and vein) identified by comparing energy intensities (see Supplementary Fig. S2 for an example).

The R-factor is the residual factor generated by the LCF tool in Athena and indicates the goodness of fit, with R-factor=∑i(data–fit)^2^/∑i(data)^2^.

^*a*^ C-Se-C compounds refer to selenomethionine (SeMet) or methylselenocysteine (MeSeCys).

### Mapping speciation of Se in hydrated roots exposed to Se(IV)

Analysis using fluorescence-XANES imaging (with an 86 energy stack) took 86 times longer than required to obtain a single elemental map with the same scan parameters. Therefore, it was necessary to evaluate whether these longer scan durations caused damage to the hydrated plant tissues. This potential beam damage was investigated by checking for changes in either elemental distributions (i.e. comparing the ‘pre-XANES survey scan’ with the ‘post-XANES survey scan’) or root morphology—no changes were evident (Supplementary Fig. S4 at *JXB* online).

For rice seedlings exposed to Se(IV), initial survey scans showed that the highest concentrations of Se in roots were in the meristem and other apical tissues (i.e. ~50–500 μm from the root apex) (Fig. 1A, B). C-Se-C compounds dominated in all tissues throughout the root cylinder (Table 2; Fig. 1C, D). Indeed, across the entire portion of root analysed using fluorescence-XANES imaging, LCF analyses indicated that uncomplexed Se(IV) only accounted for 4% of the total Se whilst C-Se-C compounds accounted for the remaining 96% (Supplementary Fig. S5A at *JXB* online). Given that the spectrum of Se(IV) has a higher intensity of the main fluorescence peak and pronounced energy shift compared with those of organic species (Supplementary Fig. S3), the contribution from selenite would be visible even at low concentrations (Supplementary Fig. S5). This was confirmed by examination of a virtual transverse transect across the root cylinder, with both the concentration of Se and the proportion present as C-Se-C compounds tending to increase with increasing distance from the root surface (Fig. 1E). Indeed, the proportion of Se present as uncomplexed Se(IV) decreased in the inner tissues, where only 1–2% was present as uncomplexed Se(IV) (Table 2; Fig. 1C, D; Supplementary Fig. S5). However, these analyses from the fluorescence-XANES imaging described above are limited because they are based upon two-dimensional representations of three-dimensional objects. For this purpose, the model of Wang *et al.* (2013*a*) was used to calculate concentrations in various individual tissues within the concentric root cylinder. The calculated concentration of uncomplexed Se(IV) was highest within the cortex (62.1 μg cm^–3^), followed by the rhizodermis (21.2 μg cm^–3^), with uncomplexed Se(IV) in the stele below the detection limit (Table 3). The calculated volumetric concentrations of C-Se-C compounds were 539 μg cm^–3^ in the stele, 355 μg cm^–3^ in the rhizodermis, and 301 μg cm^–3^ in the cortex (Table 3).

For wheat roots exposed to Se(IV), the pattern of Se accumulation and speciation was similar to that described above for rice. Specifically, the highest concentrations of Se were again found in the apical tissues (~50–1000 μm from the root apex), with concentrations being lower in the more proximal root tissues (Fig. 2). Most of the Se within the root tissues was present as C-Se-C compounds (averaged 98% across the entire region of analysis) with only 2% present as uncomplexed Se(IV). Analysis of the spatial distribution of these Se species showed that the proportion of Se present as Se(IV) was highest in the outer tissues and was lowest in the inner tissues (Table 2; Fig. 2C). Indeed, the mathematical model estimated that the concentration of uncomplexed Se(IV) decreased from 8 μg g^–1^ in the rhizodermis to ≤3.4 μg g^–1^ in the stele and cortex, with the remainder of the Se present as C-Se-C compounds (Tables 2, 23).

Interestingly, for rice roots exposed to Se(IV), some ‘hot spots’ of high Se concentrations were observed further from the root apex—these corresponded to lateral root primordia initiating from the pericycle (Fig. 3A, B). Fluorescence-XANES imaging showed that an average of 99% of the Se within these hot spots was present as C-Se-C compounds (Fig. 3D; Table 2).

### Mapping speciation of Se in hydrated roots exposed to Se(VI)

In a manner similar to that observed in Se(IV)-exposed roots, when rice roots were exposed to 1 μM Se(VI), Se appeared to move more readily into the stele, with the concentration in the stele higher than in the surrounding cortex and rhizodermis in the more proximal portions of roots (Fig. 4A). Overall, 80% of the total Se in the analysed roots was present as C-Se-C compounds, with 20% present as uncomplexed Se(VI) [but none could be detected as the intermediate, uncomplexed Se(IV); data not presented]. Interestingly, the proportion of this uncomplexed Se(VI) decreased with increasing distance from the root surface, with 31% in the outer tissues and 16% in the inner tissues (Table 2), this being evident by a decrease in the magnitude of the white line at 12.667 keV with increasing distance from the root surface (Fig. 4D). Again, use of the mathematical model indicated that at 600 μm behind the root apex, the highest concentrations of C-Se-C compounds were within the stele (632 μg cm^–3^), but the highest concentrations of uncomplexed Se(VI) were in the cortex (157 μg cm^–3^), followed by the rhizodermis (116 μg cm^–3^), and lowest in the stele (23.7 μg cm^–3^) (Fig. 4E; Table 3). Similarly, for a virtual longitudinal transect along the root, whilst concentrations of C-Se-C compounds remained relatively constant, the concentration (and hence proportion) of uncomplexed Se(VI) increased with increasing distance from the root apex (Fig. 4F), presumably due to the increased loading of this species towards the fully developed vascular tissues for transport to the shoot. For example, 8% of the Se was present as uncomplexed Se(VI) at 500 μm from the apex but 34% at 700 μm from the apex (Fig. 4F). It is noteworthy that Se concentrations in the more proximal root tissues were too low to allow for analysis using fluorescence-XANES imaging.

The results for wheat roots exposed to Se(VI) were similar to those for rice (Fig. 5). On average, a total of 9% of the Se was present as uncomplexed Se(VI) whilst 91% was present as C-Se-C compounds (data not presented). Unlike in rice roots, there was no obvious decrease in the proportion of Se present as uncomplexed Se(VI) with increasing distance from the surface of wheat roots (Table 2; Fig. 5D). However, within a virtual longitudinal transect, the concentration (and proportion) of this uncomplexed Se(VI) was again observed to increase with increasing distance from the root apex (Fig. 5F). Indeed, the concentrations of uncomplexed Se(VI) increased from 21 μg cm^–3^ (i.e. 4.1% of the total Se, at 500 μm from the apex) to 91 μg cm^–3^ (i.e. 15% of the total Se, at 1100 μm).

### Mapping speciation of Se in plant leaves

Analyses of bulk leaf tissues revealed that Se concentrations were approximately six times higher in leaves of Se(VI)-exposed plants than in those of Se(IV)-exposed plants, regardless of the plant species (Table 1). For wheat, concentrations of Se were higher within the leaf vein than in the inter-veinal tissues (Fig. 6; Supplementary Fig. S6 at *JXB* online). However, the opposite was true for rice, with Se concentrations higher in the inter-veinal tissues (Fig. 6; Supplementary Fig. S6). Interestingly, for plants exposed to Se(VI), both the leaf vein and the inter-veinal regions were dominated by uncomplexed Se(VI) (52–56% of the total Se), with C-Se-C compounds accounting for the remaining 44–48% (Table 4; Fig. 6). In contrast, no variation in Se speciation was observed in leaves of plants exposed to Se(IV) (Table 4; Supplementary Fig. S7), with almost all of the Se present as C-Se-C compounds (Table 4).

## Discussion

Although some previous studies have provided data regarding the speciation or distribution of Se within plant tissues (see Bulska *et al.*, 2006; Li *et al.*, 2008; Wang *et al.*, 2013*b*), there has generally been a division between speciation techniques (e.g. HPLC-ICP-MS, bulk XANES) and imaging techniques (e.g. μ-XRF). A comparatively common approach has been to obtain elemental maps using μ-XRF before obtaining μ-XANES spectra at specific locations of interests within such a map (for an example of this approach to the examination of Se in plant tissues, see Williams *et al.*, 2009). In this present study, elemental fluorescence maps were collected at 86 energies across the Se K absorption edge. This method allowed extraction of XANES spectra from the entire map, providing *in situ* information on the laterally resolved speciation of Se within hydrated and fresh plant tissues.

This study has demonstrated that within the root tissues, Se(VI) was efficiently reduced and converted to C-Se-C compounds, with concentrations of uncomplexed Se(VI) low even in the rhizodermis [directly exposed to the Se(VI)-containing nutrient solution]. Interestingly, uncomplexed Se(IV) was not detected in any tissues of the Se(VI)-exposed roots, indicating that the reduction of Se(VI) to Se(IV) is the rate-limiting step in the metabolism of Se(VI). Although concentrations of uncomplexed Se(VI) within the root tissues were low, this form of Se was important for translocation to above-ground tissues (TF=8.5). Once within the leaves, much of this Se remained as uncomplexed Se(VI), which accounted for 52–56% of the total Se. For Se(IV)-exposed plants, almost all of the Se was present in C-Se-C compounds (with low mobility); even within the rhizodermis and outer cortex of the root, <5 % of the total Se was present as uncomplexed Se(IV). Given that the conversion of Se(IV) to C-Se-C compounds is efficient (and that C-Se-C compounds are relatively immobile), the amount of Se translocated to the shoots was greatly decreased in these Se(IV)-treated plants (TF=0.75), the C-Se-C compounds being the only species detected in the leaf tissues.

Given that the reduction of Se(VI) to Se(IV) is the rate-limiting step in the metabolism of Se(VI), it is not surprising that tissues with higher metabolic activities (such as the root apical meristem and lateral root primordia) would accumulate more C-Se-C (Fis 1–6). Indeed, due to the greater reductive potential of these cells (Wayne, 2009), the rate of formation of C-Se-C would increase, with the concomitant decrease in the export of Se(VI), resulting in increased accumulation of Se. In less metabolically active tissues, the rate at which Se(VI) was reduced to Se(IV) would be lower, resulting not only in increased proportions of Se(VI) relative to C-Se-C, but also in lower overall concentrations of Se due to the comparatively high mobility of Se(VI) (e.g. in the stele, Figs 4, 5).

### Uptake and accumulation of Se in root tissues

Regardless of whether the Se was supplied as Se(VI) or Se(IV), the highest tissue concentrations of Se were within the root apices where the Se accumulated largely as C-Se-C compounds (Table 2; Figs. 1, 2, 4, 5). For example, spatial analysis of Se speciation revealed that when plants were exposed to Se(VI), the Se(VI) was efficiently converted to C-Se-C compounds as it was moved through rhizodermis and into the cortex, with the relative proportion of uncomplexed Se(VI) reducing with its radial transport (Table 2). Similar trends were observed for Se(IV), with the Se(IV) relatively efficiently assimilated to C-Se-C compounds and only small amounts of the uncomplexed Se(IV) observed even within the outer tissues (Tables 2, 23).

The present study showed that the conversion of Se(IV) to C-Se-C compounds is comparatively efficient, with C-Se-C compounds being the dominant species of Se in all roots examined even within the outer tissues of the root cylinder (Table 2; Figs 1, 2, 4, 5). This observation is consistent with previous studies that examined the speciation of Se in plants tissues. Using μ-XANES, Bulska *et al.* (2006) found that all detectable Se was assimilated into MeSeCys in both the roots and leaves of onion (*Allium cepa* L.) exposed to solutions containing 126.6 μM Se(IV) for 48h. Similarly, Wang *et al.* (2013*b*) used bulk XANES to examine cowpea [*Vigna unguiculata* (L.) Walp.] exposed to a toxic level of Se(IV) (20 μM) for 24h, and reported that almost all the Se was converted to organic forms. Using HPLC-ICP-MS, Li *et al.* (2008) studied root tissues of wheat grown in solutions containing 10 μM Se(IV) and found that 54% of the Se within the roots was present as organic forms.

Interestingly, for Se(VI)-exposed plants, the present results indicated that 80–91% of the total Se within roots was converted to C-Se-C compounds, with 9–20% present as uncomplexed Se(VI). However, it was not possible to detect the intermediate form of Se [i.e. uncomplexed Se(IV)], with this initial reduction from Se(VI) to Se(IV) being a critical step in the metabolism of Se(VI) (Terry *et al.*, 2000; Hawkesford and Zhao, 2007; Zhu *et al.*, 2009). These data are also in agreement with previous reports showing that the Se remained partially as uncomplexed Se(VI) in Se(VI)-exposed roots, with no uncomplexed Se(IV) observed (Bulska *et al.*, 2006; Li *et al.*, 2008; Wang *et al.*, 2013*b*). Therefore, these findings support the observation that the reduction of Se(VI) is the rate-limiting step in the metabolism of Se(VI) (de Souza *et al.*, 1998).

The data obtained here also provide some information on Se uptake and transporters. In contrast to Se(VI), comparatively little is known regarding the mechanisms involved in the uptake of Se(IV) by plant roots. This is probably due to the fact that Se(IV) is quickly assimilated to C-Se-C compounds, and indeed only low concentrations of uncomplexed Se(IV) are observed within the tissues (Table 2). Interestingly, the spatial data demonstrated that Se was largely located within the root meristem (~50–500 μm) (Fig. 1A, B) and at the primordia of lateral roots (Fig. 3A, B). This substantial accumulation suggested that either there is a bottleneck in the upwards translocation (which in this case could be the lack of a developed vascular system) or the uptake of Se(IV) involves the apoplastic pathway. Interestingly, the apoplastic pathway often occurs (i) in the root tip meristem where the Casparian strip has not yet fully formed (Baxter *et al.*, 2009); or (ii) around the lateral roots where the Casparian strip in the primary root is disturbed (Liu *et al.*, 2013).

### Long-distance translocation of Se and its accumulation in shoot tissues

The translocation of Se from the roots to the shoots appeared to depend upon the amount of Se which remained in uncomplexed inorganic forms, while the formation of C-Se-C compounds in both Se(VI)- and Se(IV)-exposed plants restricted such translocation, this being similar to that previously reported (Asher *et al.*, 1977; Zayed *et al.*, 1998; Hopper and Parker, 1999; Li *et al.*, 2008). Of particular interest in the present study, it was first noted that the TF was ~10-fold higher for Se(VI)-exposed plants than for Se(IV)-exposed plants (Table 1). In the roots exposed to Se(VI), even though the concentrations of uncomplexed Se(VI) decreased radially from the surface to inner tissues (Figs 4E, 5E), the concentrations of uncomplexed Se(VI) substantially increased longitudinally near the vascular tissue behind the apex (Figs 4F, 5F). Comparison of speciation in Se(VI)- and Se(IV)-exposed plants also supports the importance of uncomplexed inorganic Se for translocation to above-ground tissues. In plants exposed to Se(VI), ~10–30% of the Se remained as uncomplexed Se(VI) available for transport (Table 2). In contrast, in Se(IV)-exposed roots, the concentrations of uncomplexed Se(IV) were close to the detection limit. Presumably these small amounts of uncomplexed Se(IV) were actually translocated to the shoots. Indeed, C-Se-C compounds are known to have low mobility in the xylem, and, following its formation, much of the C-Se-C compounds is sequestered within vacuoles as a detoxification mechanism (Arvy, 1993; Terry *et al.*, 2000; Hawkesford and Zhao, 2007; Li *et al.*, 2008; Pyrzynska, 2009; Zhu *et al.*, 2009).

In leaves of plants exposed to Se(VI), uncomplexed Se(VI) was the dominant form of Se (Table 4; Fig. 6), thereby supporting the observation that uncomplexed Se(VI) was important for translocation. However, the proportion of uncomplexed Se(VI) did not vary across the leaf (Table 4; Fig. 6), suggesting that the rate of reduction of Se(VI) was similar in both the veins and the remainder of the leaf tissue. Again, the presence of uncomplexed Se(IV) at concentrations below the detection limit in the leaves confirmed that the reduction from Se(VI) to Se(IV) is a limiting factor in Se assimilation.

In summary, using fluorescence-XANES imaging, laterally resolved data have been provided regarding the speciation of Se within hydrated roots and leaves of wheat and rice supplied with Se(VI) or Se(IV). The Se tended to accumulate in the root apices, with C-Se-C compounds as the dominant form of Se in all roots regardless of the form of Se supplied. However, substantial differences in the uptake and laterally resolved speciation of Se were observed between Se(VI)- and Se(IV)-exposed plants. When plants were exposed to Se(VI), the reduction of Se(VI) to Se(IV) appeared to be the rate-limiting step in the formation of C-Se-C compounds. Regardless, the remaining uncomplexed Se(VI) was important for its translocation to the shoots. For plants exposed to Se(IV), almost all of the Se was efficiently converted to C-Se-C compounds in all tissues; only <5% of the Se remained as uncomplexed Se(IV), leading to only limited transport of Se to the shoot. Finally, for Se(IV)-exposed roots, substantial accumulation of Se was observed within root meristem and lateral root primordia where the Casparian strip is not fully formed or disturbed.

## Supplementary data

Supplementary data are available at *JXB* online.

Figure S1. Rice roots exposed to nutrient solution containing 1 μM Se(VI) for 1 week.

Figure S2. Leaves of wheat and rice grown in nutrient solution containing 1 μM Se(VI) for 1 week.

Figure S3. Normalized Se K-edge XANES spectra for aqueous Se standards.

Figure S4. Light micrographs and μ-XRF maps of wheat roots exposed to 1 μM Se(IV) either before or after the XANES imaging scan in order to investigate potential damage to the roots.

Figure S5. Rice (*Oryza sativa* L.) roots exposed to nutrient solution containing 1 μM Se(IV) for 1 week.

Figure S6. Areal concentration of Se in leaves of wheat (*Triticum aestivum* L.) and rice (*Oryza sativa* L.) grown in nutrient solution containing 1 μM Se(IV) for 1 week.

Figure S7. Leaves of wheat (*Triticum aestivum* L.) and rice (*Oryza sativa* L.) grown in nutrient solution containing 1 μM Se(IV) for 1 week.

Supplementary Data
