# Supplementary material for: Synchrotron-based X-ray absorption near-edge spectroscopy imaging for laterally resolved speciation of selenium in fresh roots and leaves of wheat and rice
Source: J Exp Bot. 2015 May 26;66(15):4795–806. doi: 10.1093/jxb/erv254 (PMC4507780; doi:10.1093/jxb/erv254)
Supplement: Supplementary Data [file supp_66_15_4795__index.html]

Synchrotron-based X-ray absorption near-edge spectroscopy imaging for laterally resolved speciation of selenium in fresh roots and leaves of wheat and rice — Synchrotron-based X-ray absorption near-edge spectroscopy imaging for laterally resolved speciation of selenium in fresh roots and leaves of wheat and rice — Supplementary Data 

# Synchrotron-based X-ray absorption near-edge spectroscopy imaging for laterally resolved speciation of selenium in fresh roots and leaves of wheat and rice

## Supplementary Data

Data files

- Supplementary Data - Supplementary Data
