# Supplementary material for: Synchrotron-based X-ray absorption near-edge spectroscopy imaging for laterally resolved speciation of selenium in fresh roots and leaves of wheat and rice
Source: J Exp Bot. 2015 May 26;66(15):4795–806. doi: 10.1093/jxb/erv254 (PMC4507780; doi:10.1093/jxb/erv254)
Supplement: Supplementary Data [file supp_erv254_jexbot144956_file001.pdf]

Synchrotron-based XANES imaging for laterally resolved speciation of selenium in fresh roots and leaves of wheat and rice

Peng Wang[1,*], Neal W. Menzies[1], Enzo Lombi[2], Brigid A. McKenna[1], Simon James[3], Caixian Tang[4], and Peter M. Kopittke[1]

[1] *The University of Queensland, School of Agriculture and Food Sciences, St. Lucia, Queensland, 4072, Australia*

[2] *University of South Australia, Centre for Environmental Risk Assessment and Remediation, Mawson Lakes, South Australia, 5095, Australia*

[3] *Australian Synchrotron, Clayton, Victoria, 3168, Australia*

[4] *La Trobe University, Centre for AgriBioscience, Bundoora, Victoria, 3086, Australia*

[*]To whom correspondence should be addressed. Phone: +61 7 3365 4816, Fax: +61 7 3365 1177, Email: p.wang3@uq.edu.au

Supplementary Data

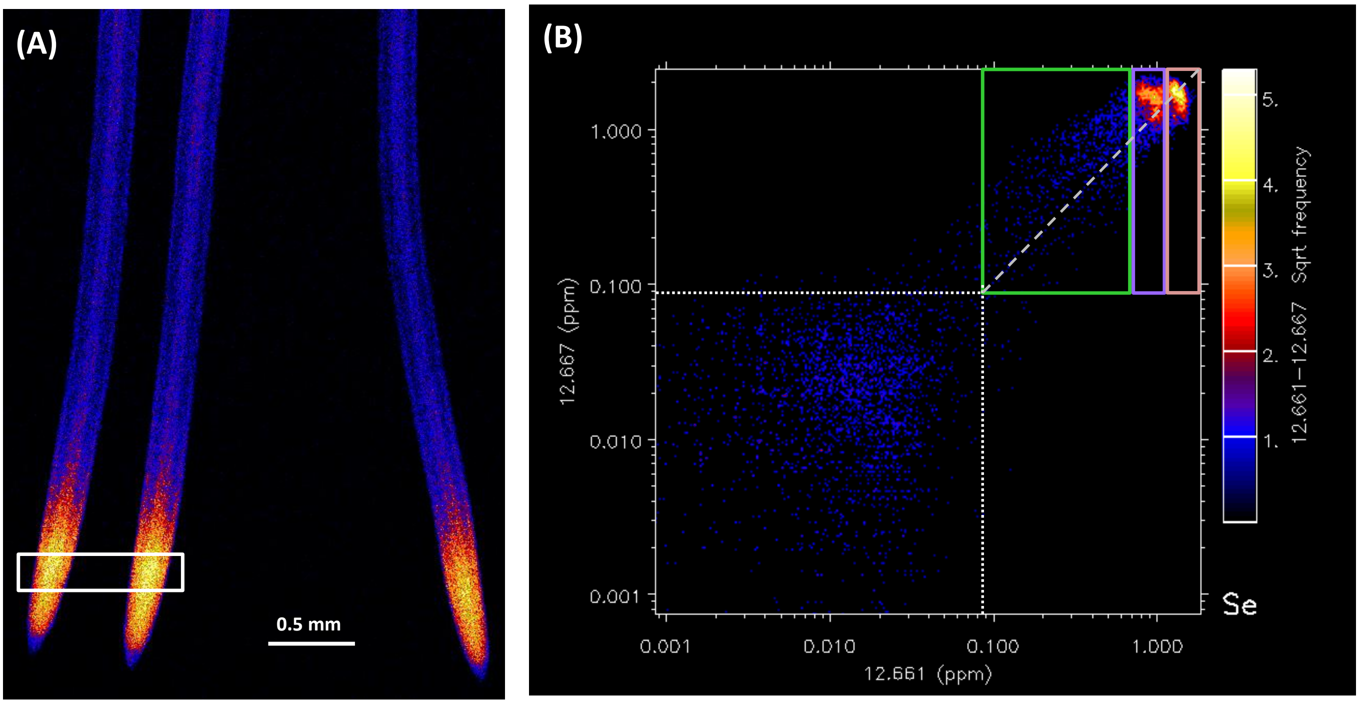

Fig. S1. Rice roots exposed to nutrient solution containing 1 μM Se(VI) for one week. (A) An elemental survey map showing total Se distribution collected in ‘pre-XANES survey scan’ followed by fluorescence-XANES imaging (‘XANES imaging scan’), with the white box indicating the area examined by XANES imaging. (B) An association plot from XANES imaging showing the relationship between energies (12.667 and 12.661 keV – these being the white lines of uncomplexed Se(VI) and organoselenium, see Supplementary Fig. S3) for all pixels, the dashed line is 1:1. The three coloured rectangles in B represent the populations of pixels highlighted in Fig. 4 from which XANES data were obtained (‘outer’, ‘middle’, and ‘inner’) – concentrations lower than those in the three coloured rectangles were determined to be background noise.

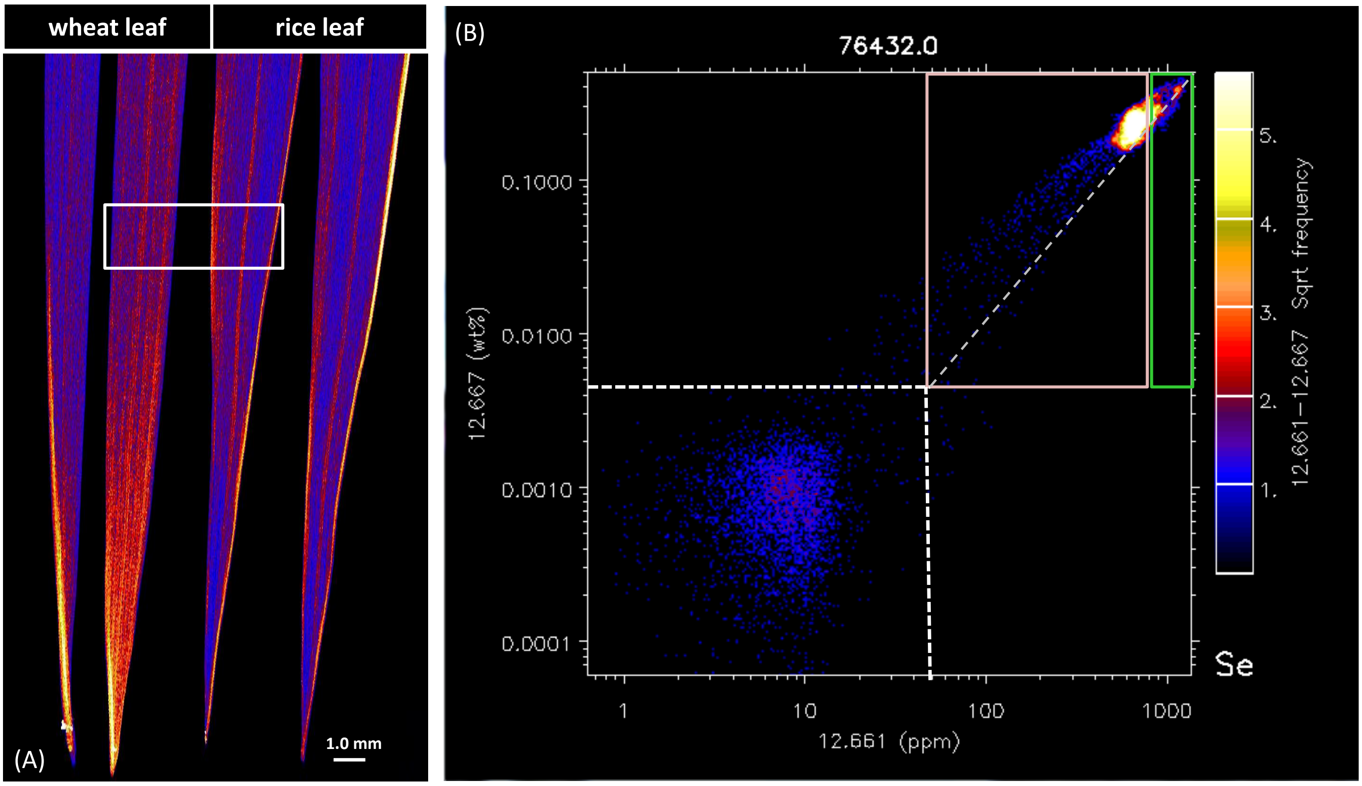

Fig. S2. Leaves of wheat and rice grown in nutrient solution containing 1 µM Se(VI) for one week. (A) An elemental survey map showing total Se distribution collected in 'pre-XANES survey scan' followed by fluorescence-XANES imaging ('XANES imaging scan'), with the white box indicating the area examined by XANES imaging. (B) An association plot from XANES imaging showing the relationship between energies (12.667 and 12.661 keV – these being the white lines of uncomplexed Se(VI) and organoselenium, see Supplementary Fig. S3) for all pixels, the dashed line is 1:1. The two coloured rectangles in B represent the populations of pixels highlighted in Fig. 6 from which XANES data were obtained ('leaf vein', and 'inter-vein') – concentrations lower than those in the three coloured rectangles were determined to be background noise.

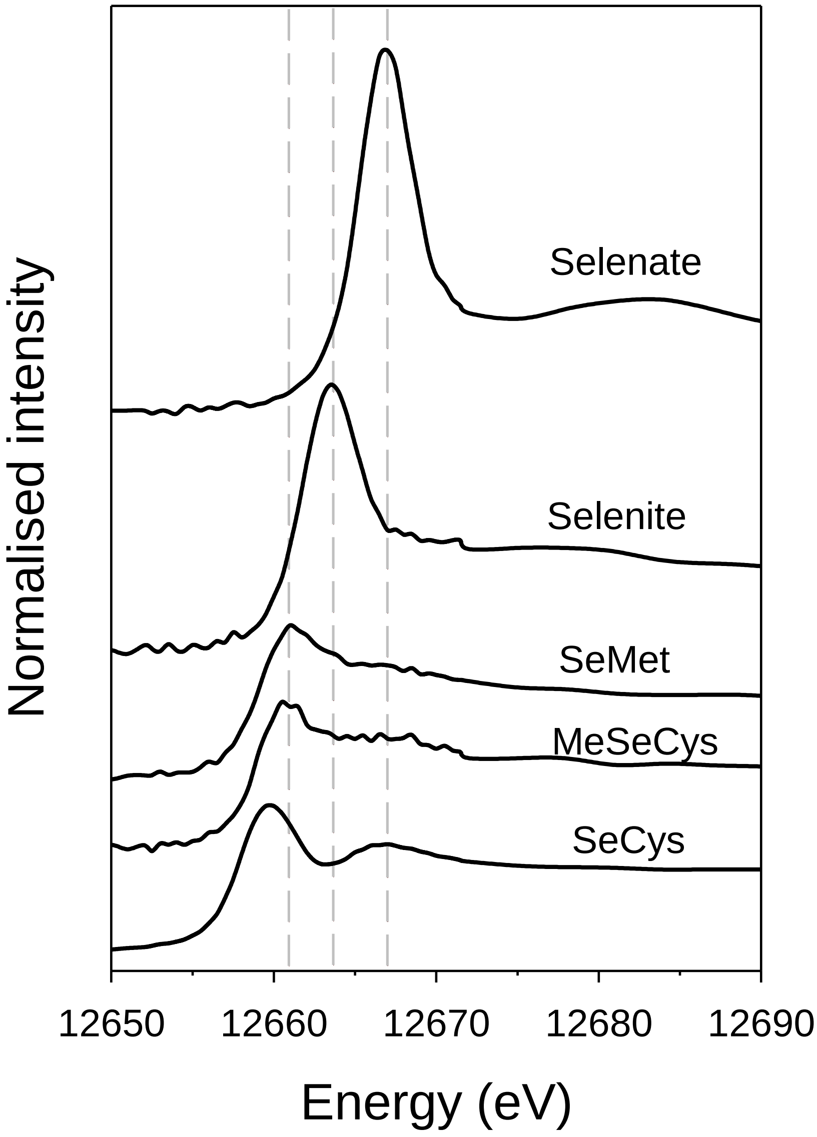

Fig. S3. Normalized Se K-edge XANES spectra for aqueous Se standards: Na_2SeO_4 (selenate), Na_2SeO_3 (selenite), selenocysteine (SeCys), selenomethionine (SeMet), and methylselenocysteine (MeSeCys).

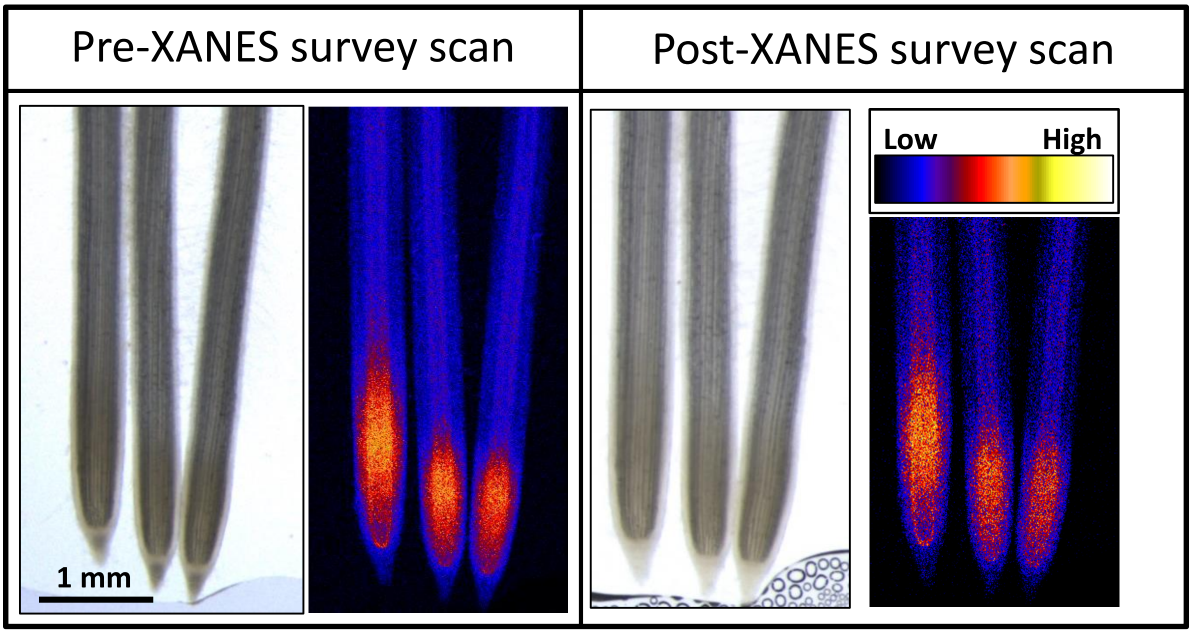

Fig. S4. Light micrographs and μ-XRF maps of wheat roots exposed to 1 μM Se(IV) either before or after the XANES imaging scan in order to investigate potential damage to the roots. The μ-XRF maps show the distribution of Se (lighter colors indicate higher concentrations). Note that the 'post-XANES survey scan' had a velocity of 1.024 mm s^{-1} (c.f. 0.512 mm s^{-1} for the 'pre-XANES survey scan') and hence the comparative quality of this scan is somewhat reduced. The scale bar applies to all panels.

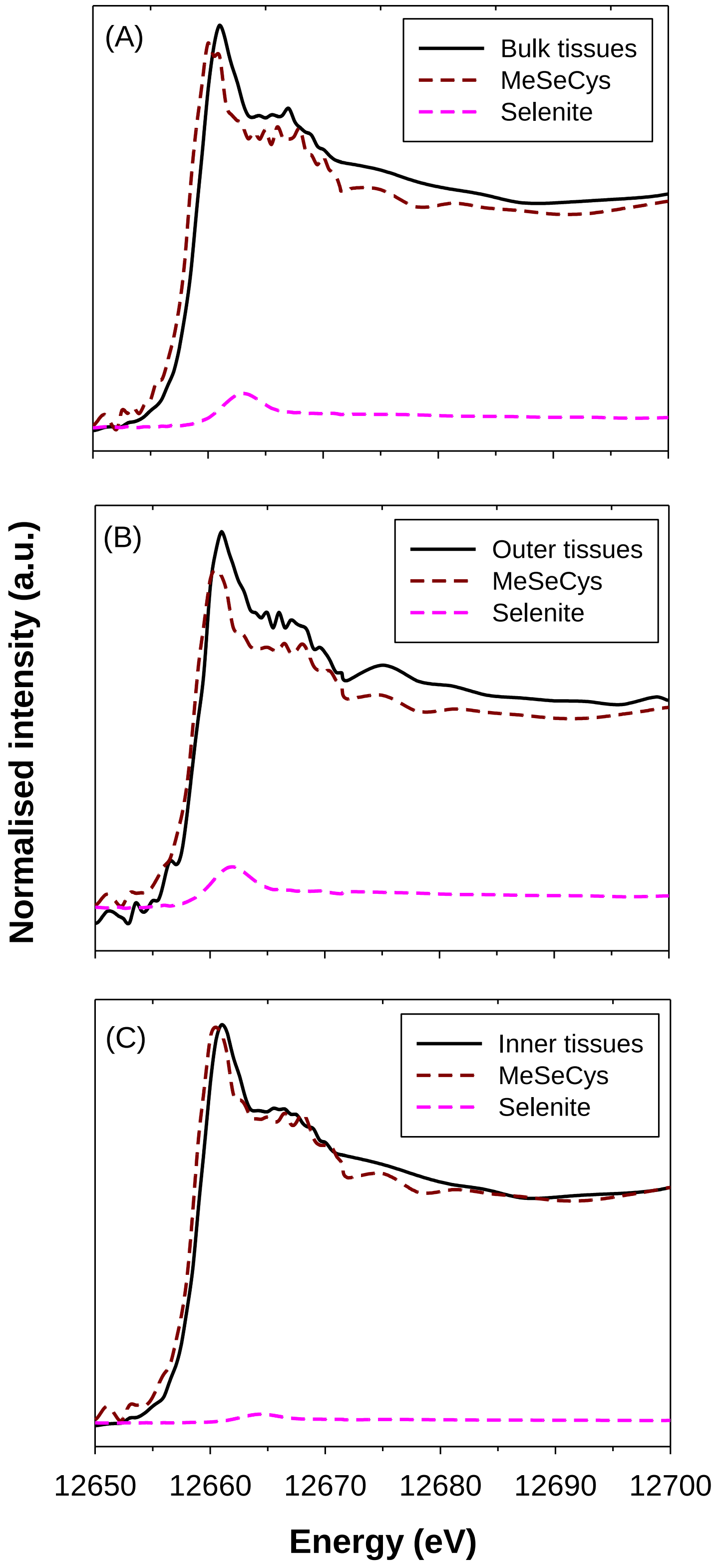

Fig. S5. Rice (*Oryza sativa* L.) roots exposed to nutrient solution containing 1 µM Se(IV) for one week. Results of performing linear combination analysis on normalized Se K-edge XANES spectra corresponding to the bulk root tissues (i.e. cross the entire portion of inner and outer tissues) and the two pixel populations 'outer' and 'inner' tissues shown in Figure 3. The data are depicted by solid black line; the MeSeCys fit component is indicated by a dashed dark red line and the selenite is indicated by dashed pink line.

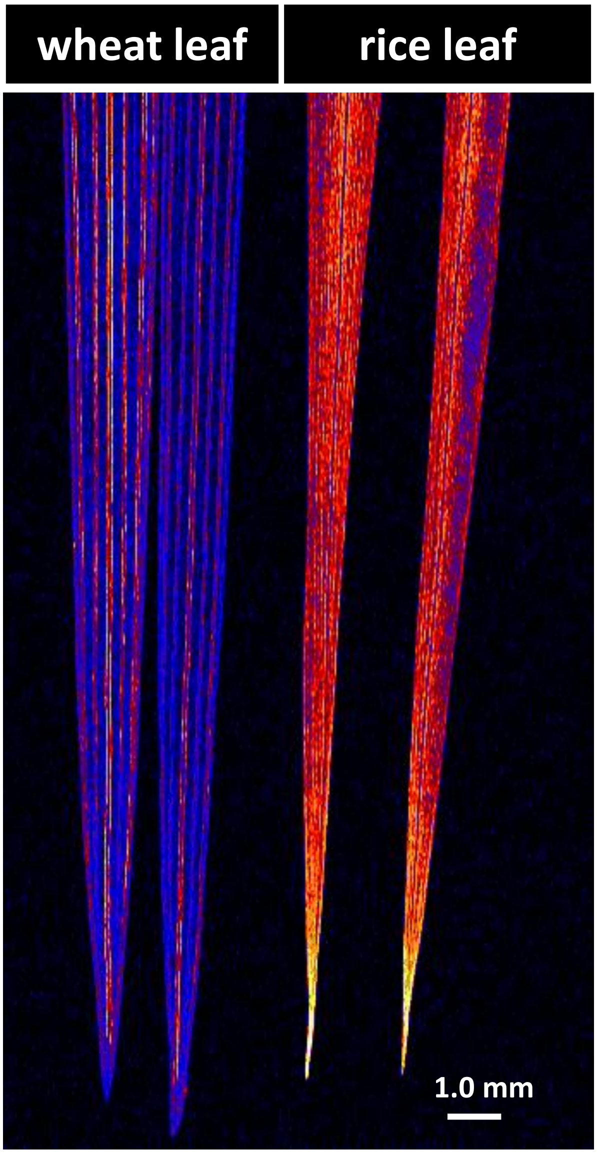

Fig. S6. Areal concentration of Se in leaves of wheat (*Triticum aestivum* L.) and rice (*Oryza sativa* L.) grown in nutrient solution containing 1 µM Se(IV) for one week. Given that that the images show areal concentrations (which are influenced by sample thickness), this image was first normalized to the Compton signal – this enabling a more accurate comparison between the veins and inter-veinal regions. The images in Fig. 6 show areal concentrations that have not been normalized according to the Compton signal.

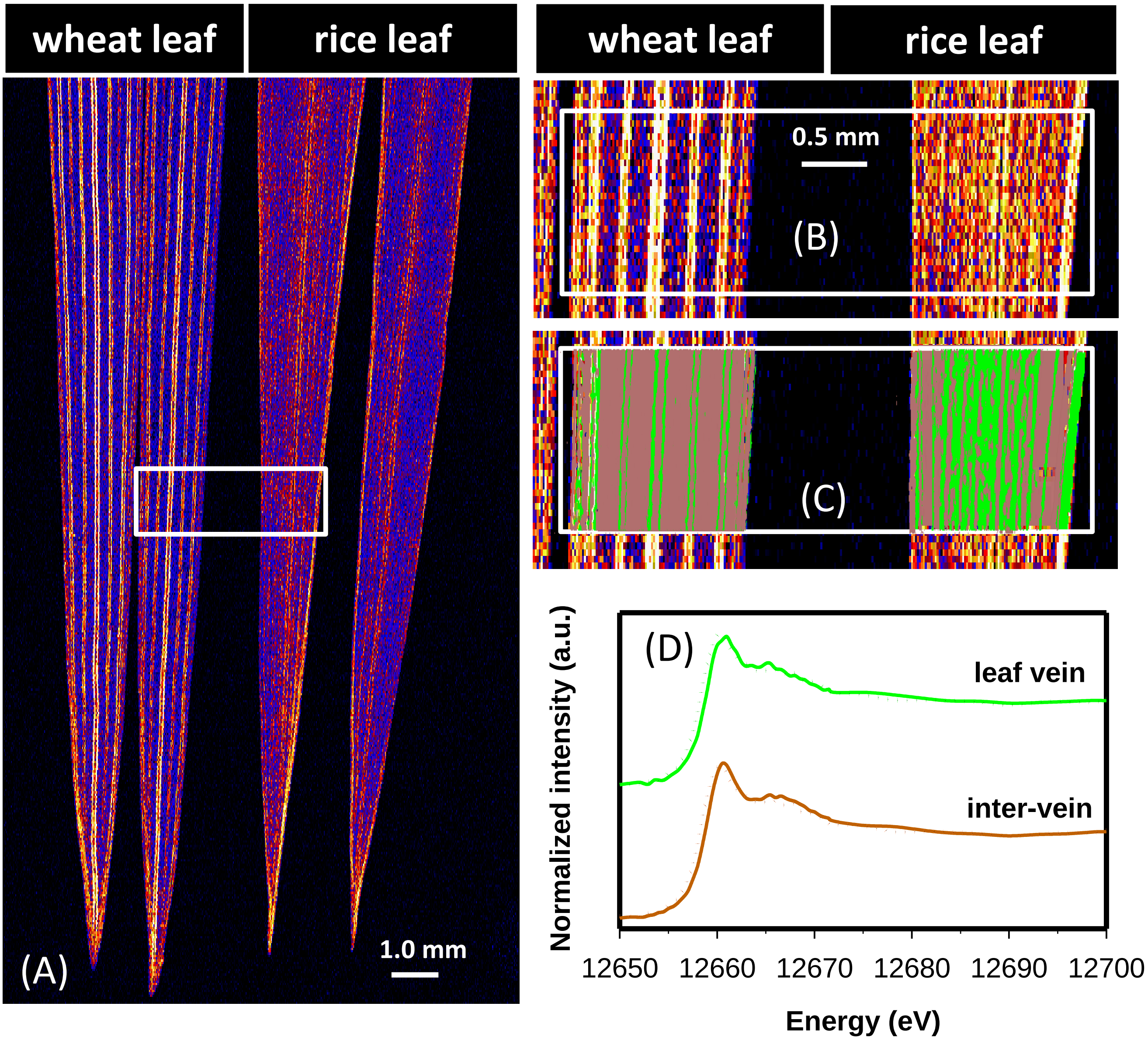

Fig. S7. Leaves of wheat (*Triticum aestivum* L.) and rice (*Oryza sativa* L.) grown in nutrient solution containing 1 μM Se(IV) for one week. (A) and (B) Elemental survey maps showing total Se distribution collected in 'pre-XANES survey scan' followed by fluorescence-XANES imaging ('XANES imaging scan'), with the white box (4.0 mm × 1.4 mm) indicating the area examined by XANES imaging. (C) The spatial distribution of two pixel-populations (leaf vein and inter-vein) identified by comparing energy intensities. (D) Normalized Se K-edge XANES spectra corresponding to the two pixel populations 'leaf vein' and 'inter-vein'.
